# Repellency of Selected Algerian-Origin Essential Oils and Hybrid Formulations Against Two Arbovirus Vectors, *Culex pipiens* s.l. and *Aedes albopictus*

**DOI:** 10.3390/insects17080823

**Published:** 2026-08-07

**Authors:** Nassima Meghazi, Abdel Madjid Benzehra, Abdenour Boumechhour, Aissam Bousbia, Rudy Megido Caparros, Frédéric Francis, Slimane Boukraa

**Affiliations:** 1 Department of Agriculture and Forestry Zoology, National Higher Agronomic School, Algiers 16004, Algeria; abdelmadjid.benzehra@edu.ensa.dz (A.M.B.); slimane.boukraa@edu.ensa.dz (S.B.); 2 Centre for Scientific and Technical Research in Physico-Chemical Analysis (CRAPC) BP 384 Bousamail, Tipaza 42009, Algeria; b.abdenour@gmail.com; 3 Laboratory of Biology, Water and Environment, Department Natural Sciences and Life, Faculty of Natural Sciences, Life Sciences, Earth and the Universe, 8 May 1945 University of Guelma, Guelma 24000, Algeria; bousbia.aissam@univ-guelma.dz; 4 Unit of Functional and Evolutionary Entomology, Gembloux Agro-Bio Tech, University of Liège, Passage des Déportés 2, 5030 Gembloux, Belgium; r.caparros@uliege.be (R.M.C.); frederic.francis@uliege.be (F.F.); 5 Laboratory of Plant Protection in Agricultural and Natural Environments, National Higher Agronomic School, Algiers 16004, Algeria

**Keywords:** mosquitoes, essential oils, *Mentha spicata*, *Ruta tuberculata*, *Lavandula latifolia*, repellency

## Abstract

Mosquitoes are significant public health insects responsible for numerous vector-borne diseases. Injudicious chemical use has developed metabolic and genetic resistance in mosquitoes. This study evaluated the repellent efficacy of five essential oils (EOs) from local plants [*Mentha spicata* I, *Ruta tuberculata*, *Mentha spicata* II, *Lavandula latifolia*, and *L. stoechas*]. *M. spicata* I and *R. tuberculata* EOs were extracted using Clavenger hydrodistillation. Simultaneously, the remaining EOs were obtained through a local extraction distillation unit. Chemical composition was identified using GC-MS. Repellency was assessed using the arm-in-cage method, by rearing *Culex pipiens* s.l. and *Aedes albopictus* in the laboratory. Additionally, mixtures containing 15% DEET, 15% IR3535, 5% vanillin, and 10% *M. spicata* I EO were tested. Both mosquito species were clearly repelled by all EOs. *M. spicata* I was most potent at 10% concentration. The complete protection time (CPT) at 10% *M. spicata* I EO was increased using blended mixtures containing 15% DEET, 15% IR35353, or 5% vanillin. Regional variations in *M. spicata* EOs were detected. This study may serve as a foundation for cosmetic manufacturers and pest control products. Developing botanical and hybrid (natural and synthetic) formulations could provide strong protection against insect bites, particularly from disease-vector mosquitoes, helping to prevent vector-borne diseases.

## Simple Summary

Mosquitoes pose a significant threat to public health as vectors of various diseases. While synthetic repellents are widely used, plant-derived essential oils (EOs) offer a safer, more eco-friendly alternative. Algeria possesses a rich but largely unexplored flora of aromatic and medicinal plants with great potential for vector control. This study aims to evaluate the repellent efficacy of five local EOs of plants extracted from two regional variants of mint (*Mentha spicata* I & II), two wild lavenders (*Lavandula latifolia* and *L. stoechas*), and *Ruta tuberculata* against two major mosquito species, *Culex pipiens* s.l. and *Aedes albopictus*. Using the arm-in-cage assay, we tested these EOs at two concentrations, both alone and in hybrid mixtures combined with DEET/IR3535/vanillin. Both mosquito species were clearly repelled by all the EOs. *M. spicata* I EO from Ghardaïa exhibited the highest bio-based repellent activity. Notably, the hybrid formulations (*M. spicata* I EO/DEET/IR3535) significantly extended the duration of complete protection time (CPT) and repellency, providing up to 600/630 min of CPT. These findings highlight the potential of local aromatic plants as sustainable raw materials for developing eco-friendly, regionally adapted, and highly effective mosquito repellents.

## 1. Introduction

Mosquitoes are significant insect pests because they are vectors of several major human diseases, including malaria, dengue, yellow fever, and West Nile Virus [1]. Over 700,000 deaths every year are caused by vector-borne diseases, of which dengue is the most common *Aedes* mosquito-borne viral disease, accounting for an estimated 40,000 deaths annually [2]. The Asian tiger mosquito *Aedes albopictus* (Skuse, 1894) is considered one of the 100 most invasive species in the world and is increasingly being recognized as a significant vector [3]. It is a persistent day-biter and a common pest of humans, mammals, and birds [4]. Its rapid global spread has been primarily driven by human mobility and the transport of used tires [5], along with global trade and climate change. These factors have facilitated its introduction and establishment beyond its native range, increasing the disease burden of arboviruses [6]. In August 2010, *Ae. albopictus* was first identified in Algeria, in the region of Tizi Ouzou, marking its first appearance in the Maghreb [7]. Since then, the species has become widespread in several areas, with citizen reports playing a key role in early detection. The allergic skin reaction caused by its bites has raised public health concerns regarding its vector potential and adaptability [8,9,10]. The common mosquito, *Culex pipiens* s.l. (Linnaeus, 1758), is a night-biting species. It is considered one of the most abundant and widespread mosquitoes in Algeria [11,12,13,14] and a primary vector of West Nile Virus disease [10], as well as a potential vector of Rift Valley Fever virus [15].

In addition to managing mosquitoes through insecticides, a frequently recommended strategy for mitigating mosquito bites and nuisance is individual personal protection [4,16], including topical and spatial repellents. Mosquito repellents are substances that interfere with the feeding habits of hematophagous mosquitoes, protecting against insect bites [17]. The DEET (N,N-diethyl-meta-toluamide) represents the most extensively utilized synthetic repellent, followed by Picaridin [1-piperidinecarboxylic acid 2-(2-hydroxyethyl)-1-methylpropylester, or KBR 3023] [18]. DEET is widely recognized for its effectiveness and safety profile, though misuse of these substances can negatively impact the skin, lungs, airways, and nervous system [18]. For more than 50 years, it has served as the foundation for a wide range of commercially licensed formulations with DEET concentrations ranging from 10% to 80% [19]. Furthermore, other synthetic repellents, such as IR3535 (Ethyl Butylacetylaminopropionate), have demonstrated significant potential in providing extended protection against mosquito bites [20], thereby reducing the risk of disease transmission and human exposure to hematophagous bites during outdoor activities [21].

Driven by increasing consumer demand for environmentally friendly products, current research focuses on identifying natural compounds that are effective against mosquitoes, with the ultimate goal of mitigating the transmission of mosquito-borne diseases [22]. The use of bio-insecticides derived from botanical or plant-based materials is an ideal substitute because they have minimal negative impacts on the environment and human health [22]. A significant distinction between synthetic and natural repellents lies in the duration of their repellent action [23]. However, according to Koul et al. [24], research suggests that nepetalactone, a component of the essential oil from the catnip plant (Nepeta cataria), is remarkably effective, being 10 times more potent than DEET. This indicates that only one-tenth of the nepetalactone amount is sufficient to match the efficacy of DEET. Previous studies have demonstrated the significant relevance of essential oils and plant-based extracts [25], including their antiseptic, antibacterial, antiviral, and antioxidant effects [26]. Essential oils such as lemongrass, eucalyptus, rosemary, clove, and thyme possess known insecticidal properties. Peppermint is an efficient deterrent against pests like ants, flies, lice, and moths, whereas spearmint and basil are effective in repelling flies [24]. There are only a few commercially available plant-based repellents. However, Citronella oil emerged as an early insect repellent in 1908 and remains a common component of insect-repellent products today [27], while p-menthane-3,8-diol (PMD) is a monoterpene and a plant-based organic compound used to deter mosquitoes [16]. It is referred to as the residual material that remains following the distillation of Lemon eucalyptus essential oil from the leaves [28].

According to Dobignard & Chatelain in the work of Meddour et al. [29], Algeria is renowned for its floristic richness, which includes over 3951 native taxa (species and subspecies), of which 290 are endemic. This botanical diversity serves as a valuable reservoir of medicinal and aromatic plants, specifically prized for their essential oils. Extensive research has explored the significant biological properties of essential oils [30]. However, the repellent efficacy of indigenous Algerian plant essential oils against mosquitoes remains largely uncharted. Despite a few studies on the insecticidal and larvicidal activities of essential oils against mosquito larvae [31,32,33,34], their repellent effects on adult mosquitoes require further investigation. Fundamentally, the main role of EO compounds is to protect plants against phytophagous insects [35], and they have been shown to have repellent and deterrent effectiveness against mosquitoes and other biting insects. Lamiaceae and Rutaceae are among the main families investigated for their potential to repel mosquitoes [36]. To the best of our knowledge, this is the first report on the repellent efficacy of *Ruta tuberculata* Forssk. (Sapindales: Rutaceae) and *Lavandula latifolia* Medik. (Lamiales: Lamiaceae) essential oils (EOs) against *Cx. pipiens* s.l. and *Ae. albopictus*; research on the repellent potency of the wild-growing lavender species against adult female mosquitoes remains scarce in the literature. Therefore, the primary objective of this research is to evaluate the repellent properties of EOs from selected Algerian plants against two mosquito species, *Cx. pipiens* s.l. and *Ae. albopictus*, both of which are considered potential arbovirus vectors. Specifically, this study focuses on two specimens, *Mentha spicata* L. (Lamiales: Lamiaceae) I and *R. tuberculata*, sourced from Ghardaïa, in addition to commercially available EOs, including *M. spicata* II from El Bayadh and two species of wild lavender, *Lavandula stoechas* L. and *Lavandula latifolia*. Moreover, comparing these EOs to a benchmark is necessary to assess their potential use as mosquito repellents. Hence, DEET and IR3535 are employed as positive controls in this study. Finally, several combinations combining vanillin, DEET and IR3535 with the EO exhibiting the highest repellence were assessed in order to extend its complete protection time.

The findings aim to identify promising plant-derived compounds for developing effective mosquito repellents, providing a basis for botanical and hybrid formulations to protect against vector-borne diseases.

## 2. Materials and Methods

### 2.1. Plant Materials

Fresh *Mentha spicata* I specimens were collected from a mint cultivation site in Ghardaïa province, southern Algeria. Simultaneously, the dried aerial parts of *Ruta tuberculata* were sourced commercially from a traditional medicinal herb supplier in the same area. The identification was performed by a plant taxonomist at the Botany Department of the Higher School of Agronomic Science in Algiers. The pure EOs of *M. spicata* L. II, *L. latifolia*, and *L. stoechas*, as well as their Certificates of Analysis (COA), were obtained from Sarl ZIPHEE.BIO (W. Bouira 10016, Algeria), an Algerian company specializing in EO production (Table 1). It is noteworthy that *M. spicata* and *M. viridis* are synonymous and refer to the same species. However, we will refer to the spearmint procured from Ghardaïa as *M. spicata* I, whereas *M. spicata* II pertains to the spearmint originating from El Bayadh (Table 1 and Table 2).

### 2.2. Isolation of Essential Oils

The essential oils (EOs) extracted from *M. spicata* I and *R. tuberculata* were acquired via hydro-distillation with a Clevenger-type apparatus. For this, 100 g of dried plant material was placed in water and extracted for 3 h. Following this, the EOs were dehydrated with anhydrous sodium sulfate and stored in a refrigerated environment for future use. According to Barbouchi et al. [37], the yield of EOs was determined using this formula:

Y_HE_ (%) = (W_2_/W_1_) × 100

with:

Y_HE_ (%): Yield of the EO (%)

W_1_: weight of the dried plant material in grams.

W_2_: weight of the EO in grams.

### 2.3. Essential Oils’ Analysis

The main analytical technique used to identify the chemical constituents of both *M. spicata* I and *R. tuberculata* EOs is gas chromatography–mass spectrometry (GC-MS). The EOs’ chemical composition was obtained using a 6890-Plus Gas Chromatograph coupled to a 5973-Mass Spectrometer (Agilent, Santa Clara 95051, CA, USA). For this, 0.2 µL of the sample was injected in split mode 50:1. The injection compartment is held at 250 °C, the transfer line at 260 °C, the MS source at 230 °C, and the quadrupole at 150 °C. The EI filament current was set to 70 eV. A capillary column (30 m × 0.25 mm × 0.25 µm), coated with 5% phenyl/95% methyl polysiloxane, was heated according to the following temperature program: 8 min at 60 °C, 2 °C/min to 250 °C, and 10 min at 250 °C. Data were analyzed using ChemStation (MSD Chemstation v. D.02.00.275) and AMDIS software (Amdis_32 v. 2.73), and identification was based on comparing mass spectra with the NIST 2.4 and Wiley 9 databases, as well as on retention indices [38].

### 2.4. Sources of Mosquito Strains

This study employed two mosquito strains, *Cx. pipiens* s.l. and *Ae. albopictus*, that originated from urban neighborhood of Algiers and surrounding areas. Various potential breeding sites were identified and randomly selected. Specifically, *Cx. pipiens* s.l. larvae were collected from underground sites and flooded basement rooms located in Derguana province, whereas *Ae. albopictus* larvae were collected from artificial and natural water-holding containers in the gardens of the National Higher Agronomic School in Algiers.

### 2.5. Mosquito-Rearing Techniques

The collected samples of both mosquito species were immediately transported to the zoology laboratory at the Higher National Agronomic School (Algiers). Rearing procedures followed the general guidelines of the World Health Organization [39] with minor modifications. Larvae were reared in open plastic trays with tap water to a depth of 2.5 cm and fed with fish diet. To prevent scum formation on the water surface and regulate the larval population density, the water was refreshed daily. Pupae were filtered into a separate plastic tray filled with water and transferred to wooden cages for adult emergence. These colonies were maintained in an insectary under controlled conditions of a stable temperature (27 ± 1 °C), relative humidity (70 ± 5%), and a 16:8 (light/dark) photoperiod. Adult mosquitoes were held in mesh cages measuring (30 × 40 × 30 cm) with a front-access fabric sleeve. Both sexes were maintained in the cages to allow for mating. Adult mosquitoes were fed a 10% sugar solution using soaked cotton balls placed in the cages. Females of the autogenous mosquitoes of *Cx. pipiens* s.l. (potentially *molestus* strain) species were able to lay their eggs without any blood meal, while *Ae. albopictus* females were fed on three-day-old chicks to obtain a blood meal. Eggs of *Ae. albopictus* were collected on moist filter paper placed inside the oviposition cups with a fine brush, and then transferred to water-filled plastic trays for hatching.

### 2.6. Mosquito Repellency Bioassay

**Figure 1:**
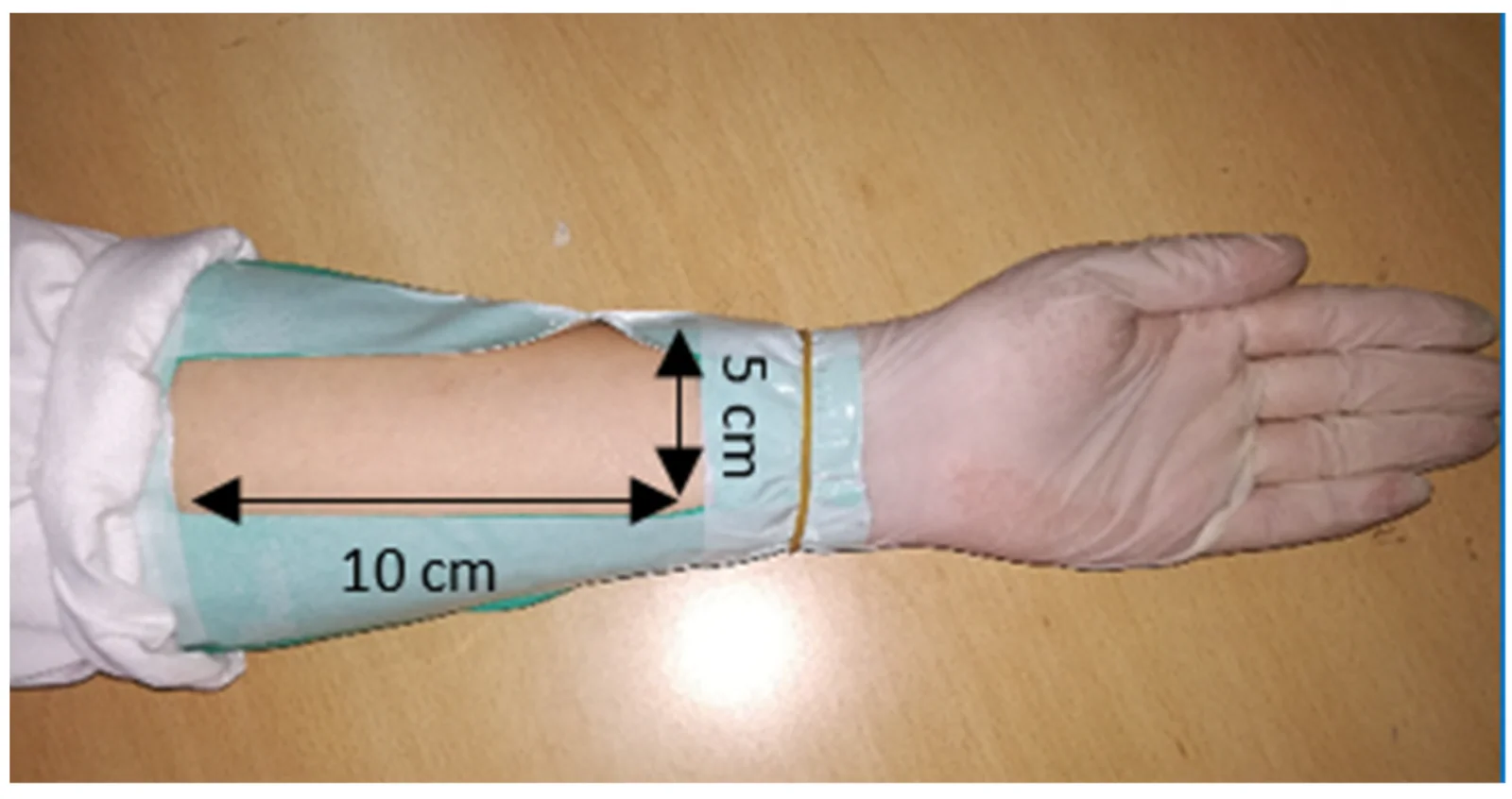
Experimental setup for the volunteer’s arm displays a 50 cm^2^ exposed area. Human skin is subjected to repellency bio-tests using the screened EOs at 5% and 10% concentrations, as well as a positive control at 15% DEET and 15% IR3535.

The experimental design used to assess the repellent activities of the extracted and commercially available EOs against *Cx. pipiens* s.l. and *Ae. albopictus* adult females was obtained using the arm-in-cage method (AIC). The objective of this test was to estimate the complete protection time (CPT) of the repellent substances. The CPT is the time elapsed between the application of the repellent and the first mosquito bite. A second bite within a 30-min interval must confirm the first bite. We refer to a bite as the insertion of a mosquito’s proboscis into a volunteer’s skin while probing for blood. The bioassays were conducted according to the WHO [39] recommendations with minor modifications. The arm area used in the repellency tests was 50 cm^2^. It was prepared by cutting out a rectangular area (5 × 10 cm^2^) on the inner forearm below the wrist, using an elbow-length polyethylene glove (Figure 1).

Before starting the repellency bioassays, or using the sleeved arms as controls, the participant’s forearms were washed with unscented soap. In our case, we applied raw Marseille soap without perfume to the forearm, which was then carefully rinsed and dried, and then rinsed with a 70% ethanol solution to prepare for the laboratory research.

Participants refrained from smoking and from using scented items such as cologne, perfume, and deodorant for at least 12 h before and during the tests. A polyethylene sleeve protected the untreated part of the forearm and hand, and a latex glove was worn to prevent mosquito bites (Figure 1). Participant inclusion was achieved through informed consent. Tests were conducted on equal numbers of female and male human volunteers (*n* = 6) aged 18 to 55, who are among candidates with no sensitivity to mosquito bites.

A series of dilutions (%*v*/*v*) of the five EOs in 70% ethanol were carried out [39]. Solutions were formulated at two concentrations: 5% and 10% *v*/*v*. Additionally, DEET and IR3535 were used as positive controls, each at 15% *v*/*v* (Table 3). A supplementary solution was also prepared by incorporating vanillin, which, although not inherently repellent to mosquitoes, acts as a fixative that enhances the persistence and effectiveness of repellents [4].

To assess the readiness of female mosquitoes to bite, an untreated control forearm of a volunteer was exposed to a test cage for 3 min, and the number of landings and/or bites was recorded. The mosquitoes were blown from the hand before any blood was taken. The cages contained 5–7-day post-emergence, non-bloodfed, active host-seeking females. The density of each species in the cage was regulated; 100 *Cx. pipiens* s.l. females were released into the test cage, whereas only 60 *Ae. albopictus* female mosquitoes were released. The glucose solution was removed 12 h before the bioassays.

The exposed area of the volunteer’s forearm was treated with 0.1 mL of 5% and 10% *v*/*v* dilutions of each substance, and the pipette tip was used to disperse the material uniformly. Sleeved forearms treated with repellent substances were exposed to female mosquitoes in the cages for at least three minutes; exposure was repeated three times and continued every 30 min until the first bite was recorded. A second consecutive bite was necessary to ensure that the substances no longer protected; at this moment, the test was stopped and the protection time was recorded. If no additional confirming bite was recorded, testing of the treated arm continued until a confirmed bite was recorded. The testing period lasted up to 8 h depending on the efficacy. The total number of mosquitoes biting and/or landing on control and treatment areas was recorded continuously throughout CPT. The mean landing and/or biting rate for the test was calculated from a series of three readings, each three minutes long.

For comparison, repellency level, and the landing and biting rates were calculated using the formulas below [48,49,50]:

$$R(\%)=\frac{\left(C-T\right)}{C}\times 100\%$$

where:

R: repellency level R%.

C: mean number of mosquitoes landing and/or biting on the control arm (control).

T: mean number of mosquito landing and/or biting on the treated arm.

Landing (%) = (L/100) × 100%

where:

L: represents the total number of mosquitoes landing by the end of the test. The test was carried out 3 times per sample.

‘100’ is the total number of adult *Cx. pipiens* s.l. females present in the cage.

Landing (%) = (L/60) × 100%

‘60’ is the total number of adult female *Ae. albopictus* present in the cage.

Biting (%) = (B/100) × 100%

where:

B: represents the total number of mosquitoes biting by the end of the test. The test was carried out 3 times per sample.

‘100’ is the total number of adult *Cx. pipiens* s.l. females present in the cage.

Biting (%) = (B/60) × 100%

‘60’ is the total number of adult female *Ae. albopictus* present in the cage.

### 2.7. Statistical Data Analysis

All statistical analyses and graphical representations were performed using R software version 4.5.1 [51]. The number of mosquito landings recorded on negative control and sample-treated hands was compared using an unpaired Student’s *t*-test. Differences in complete protection time (CPT) among the tested substances were evaluated using the non-parametric Kruskal–Wallis test. When the overall test was significant, pairwise comparisons were performed using Dunn’s post hoc test with Holm’s correction for multiple testing. Statistical significance was set at *p* < 0.05.

## 3. Results

### 3.1. Yield of Essential Oils

The extraction process revealed clear differences in essential oil yields between the two extracted species, *M. spicata* I and *R. tuberculata,* sourced from the same region (Ghardaïa). The percentage yield of *M. spicata* I EO was higher (1.40%) than that obtained from *R. tuberculata* (0.14%). Indeed, the fresh aerial parts of the spearmint plant were the most oil-rich, unlike the dried shoots plants of *R. tuberculata*.

### 3.2. Chemical Characterization of the Extracted Essential Oils

The chemical composition of *M. spicata* I EO from Ghardaïa revealed 33 compounds, accounting for 98.6% of the oil (Table 4). The most abundant compounds were monoterpenes (48.3%) and oxygenated monoterpenes (monoterpenoids) (46.6%). (S)-(+)-Carvone was identified as a major component present in *M. spicata* I EO (44.2%), followed by limonene (36.8%), D-limonene (3.0%), beta-pinene (1.6%), and alpha-pinene (1.5%).

A total of 48 compounds representing 99.8% of *R. tuberculata* EOs were identified, which are listed in Table 5. The dominant compounds were 2-undecanone (45.3%) and β-phellandrene (8.8%), followed by trans-2-menthenol, germacrene B, cis-2-menthenol, and piperitone, which accounted for 5.4%, 4.9%, 3.6%, and 3.2%, respectively.

### 3.3. Mosquito Repellency of Individual Essential Oils (EOs)

**Figure 2:**
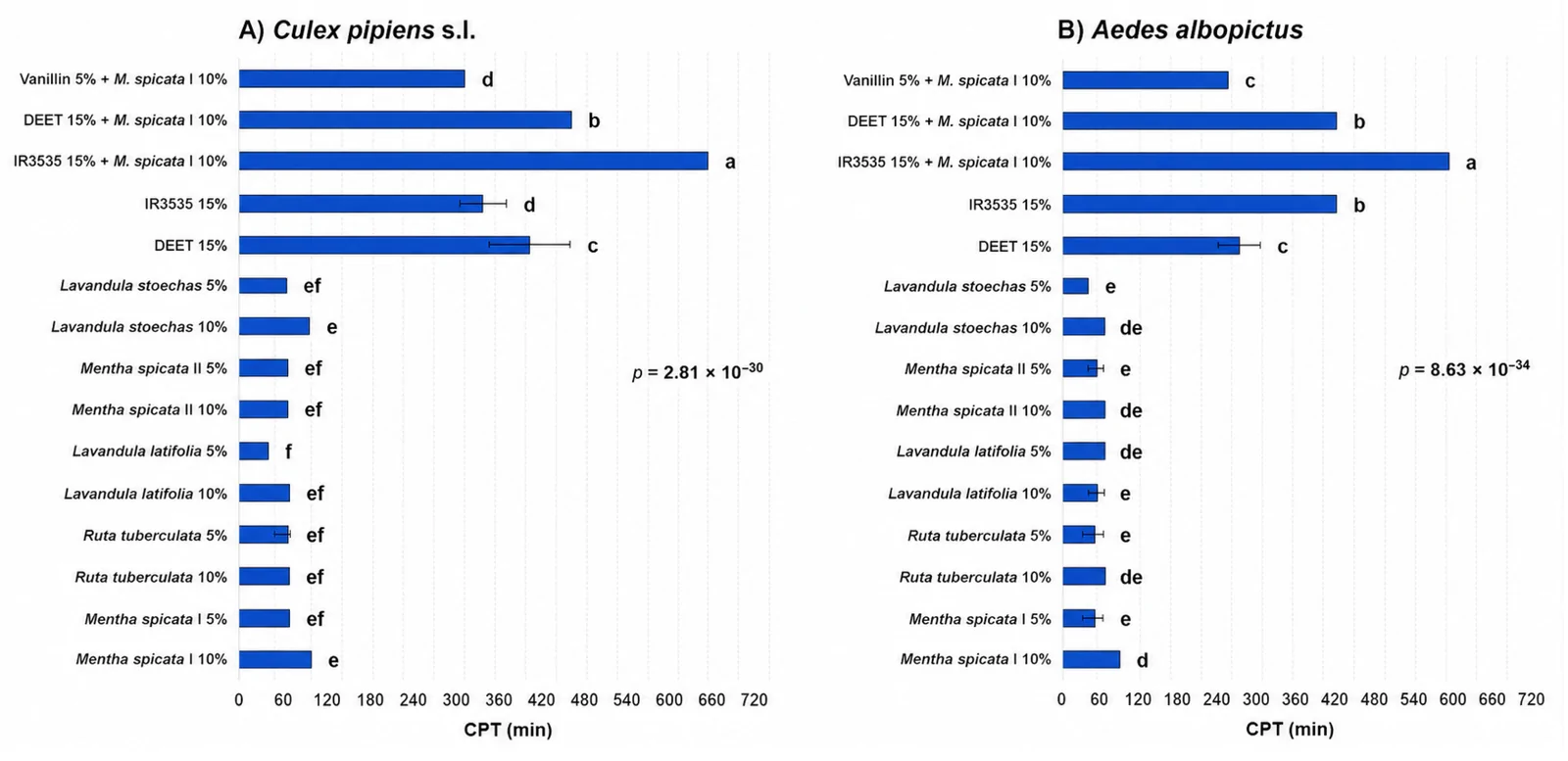
(**A**) Mean complete protection time (CPT) measured with the mosquito arm-in-cage method (AIC) for the different substances evaluated against *Culex pipiens* s.l. females. (**B**) Mean complete protection time (CPT) measured with the mosquito arm in cage method (AIC) for the different substances evaluated against *Aedes albopictus* females. Values are presented as mean ± SD (*n* = 3). Different letters on bars show significant difference (*p* < 0.05).

**Figure 3:**
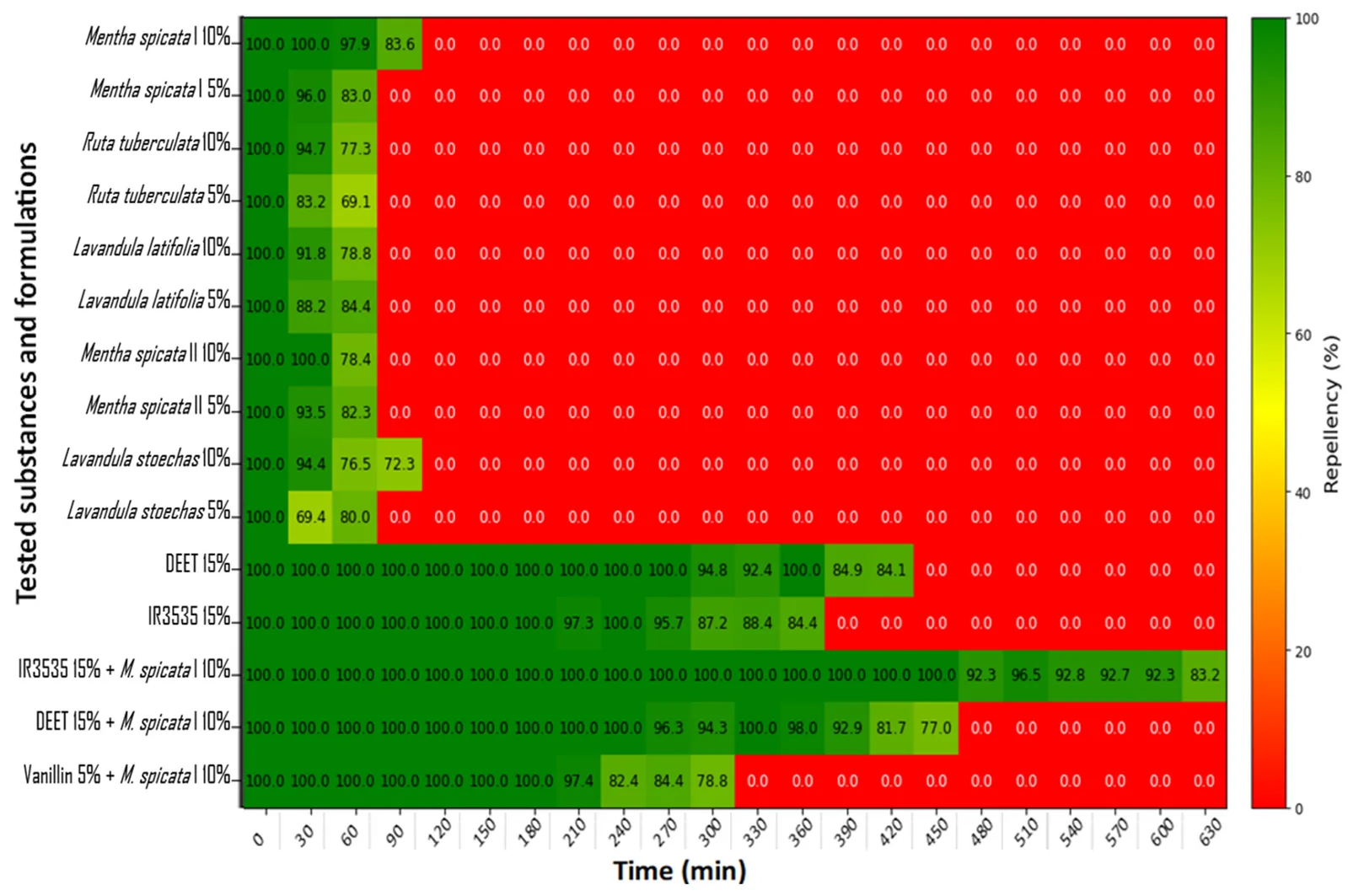
Heatmap of repellency levels (R%) of tested EOs and mixtures against *Culex pipiens* s.l.

**Figure 4:**
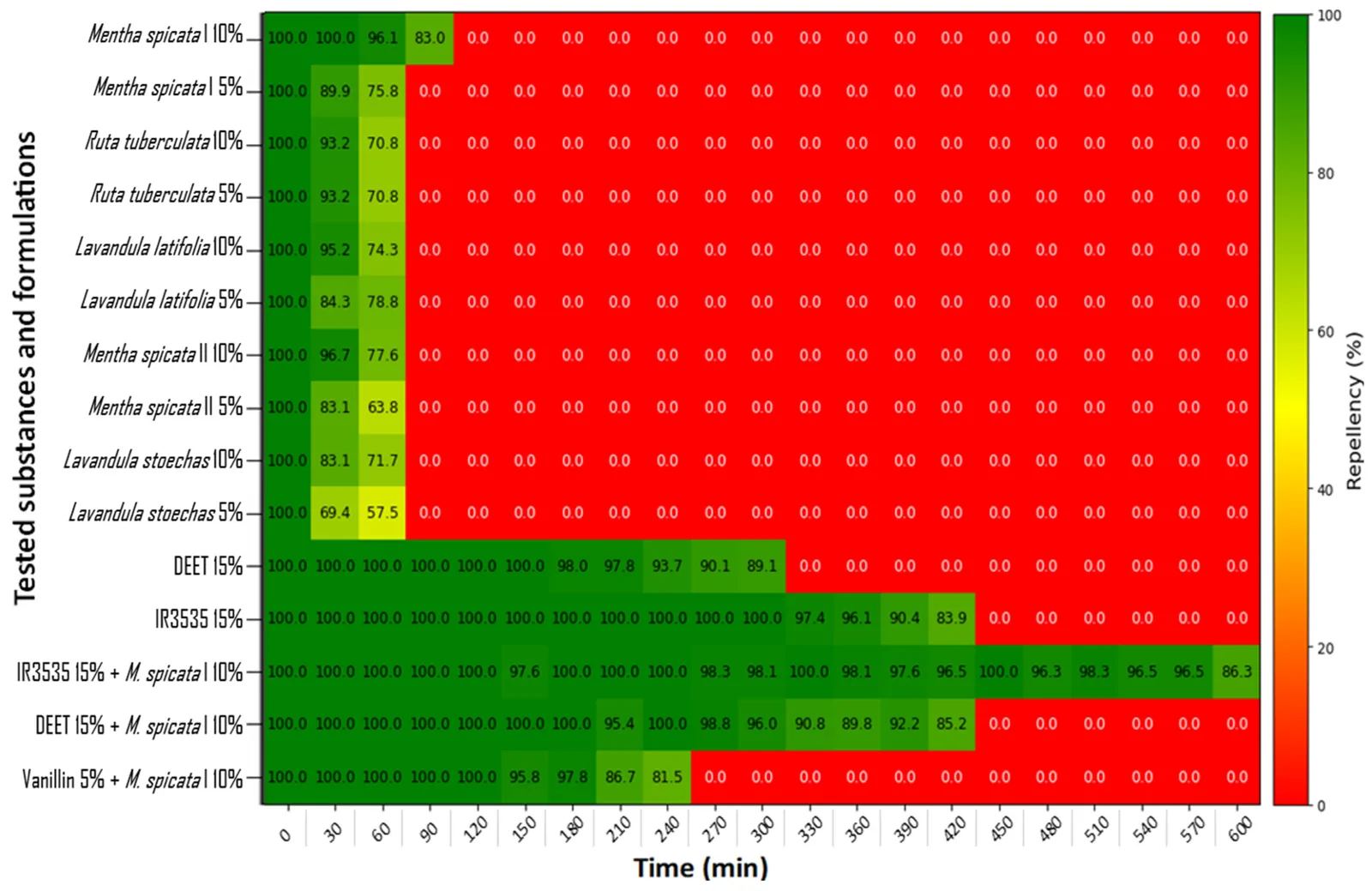
Heatmap of repellency levels (R%) of tested EOs and mixtures against *Aedes albopictus*.

The repellent effect of all tested EOs on both female mosquito species *Cx. pipiens* s.l. and *Ae. albopictus* was observed at different and significant levels (Figure 2). All the tested EOs, including *M. spicata* I, *R. tuberculata*, *M. spicata* II, *L. stoechas*, and *L. latifolia*, demonstrated more than 70% repellency against *Cx. pipiens* s.l., and achieved up to 60% repellency towards *Ae. albopictus*, at the tested doses of 5% and 10% (*v*/*v*) (Figure 3 and Figure 4).

Significant differences were observed in the repellency of *M. spicata* I 10% against the two mosquito species (Figure 2). This EO exhibited 100% repellency for 30 min, compared with the positive controls DEET and IR3535 (Figure 3 and Figure 4). *M. spicata* I and *M. spicata* II EOs at 10% concentration displayed similar repellency against *Cx. pipiens* s.l. over 30 min. However, their repellency levels decreased over time. They reached 83.6% and 78.4%, respectively, over 60 min (Figure 3). Furthermore, over a 90 min period, the repellent effectiveness of *L. stoechas* EO was the highest, with a significant difference (*p* < 0.05) compared to *M. spicata* II, *L. latifolia* and *R. tuberculata* EOs, along with *M. spicata* I EO (Figure 2).

*M. spicata* I demonstrated complete repellent efficacy against *Ae. albopictus* mosquitoes at 10% concentration within a 30-min timeframe. *L. latifolia* EO (10%), when freshly applied, exhibited 95.2% repellency after 30 min, which subsequently dropped to 74.3% after 60 min. Furthermore, *R. tuberculata* EO showed 93.2% repellency at both 10 and 5% concentrations, which decreased over time to 70.8% within 60 min of CPT. *M. spicata* II and *L. stoechas* showed superior repellency, lasting more than 30 min, achieving 96.7% and 83.1% at 10% concentration, respectively. *M. spicata* I EO sustained repellency for 90 min, achieving 83%, indicating a statistically significant difference (*p* < 0.05) (Table 6 and Table 7; Figure 2).

### 3.4. Mosquito Repellency of Different Mixtures

Among the five EOs, the one with the higher individual repellent properties was selected for blending experiments. The mixture of 10% *M. spicata* I EO with 15% IR3535 provided the longest complete protection time (CPT) against both mosquito species, surpassing the repellent efficacy of the positive control, 15% DEET, and 15% IR3535 tested individually. Notably, CPT of 630 min against *Cx. pipiens* s.l. and CPT of 600 min against *Ae. albopictus* exceeded the 420-min CPT for DEET 15% toward *Cx. pipiens* s.l. and 420 min CPT of IR3535% against *Ae. albopictus* (as illustrated in Figure 2).

The repellency of *M. spicata* I EO from Ghardaïa at a concentration of 10% was clearly enhanced by adding 5% vanillin. Specifically, this mixture provided a CPT of 300 min against *Cx. pipiens* s.l. and 240 min against *Ae. albopictus* (Table 6 and Table 7; Figure 2).

### 3.5. Mosquito Behavior Towards Repellent Substances

**Figure 5:**
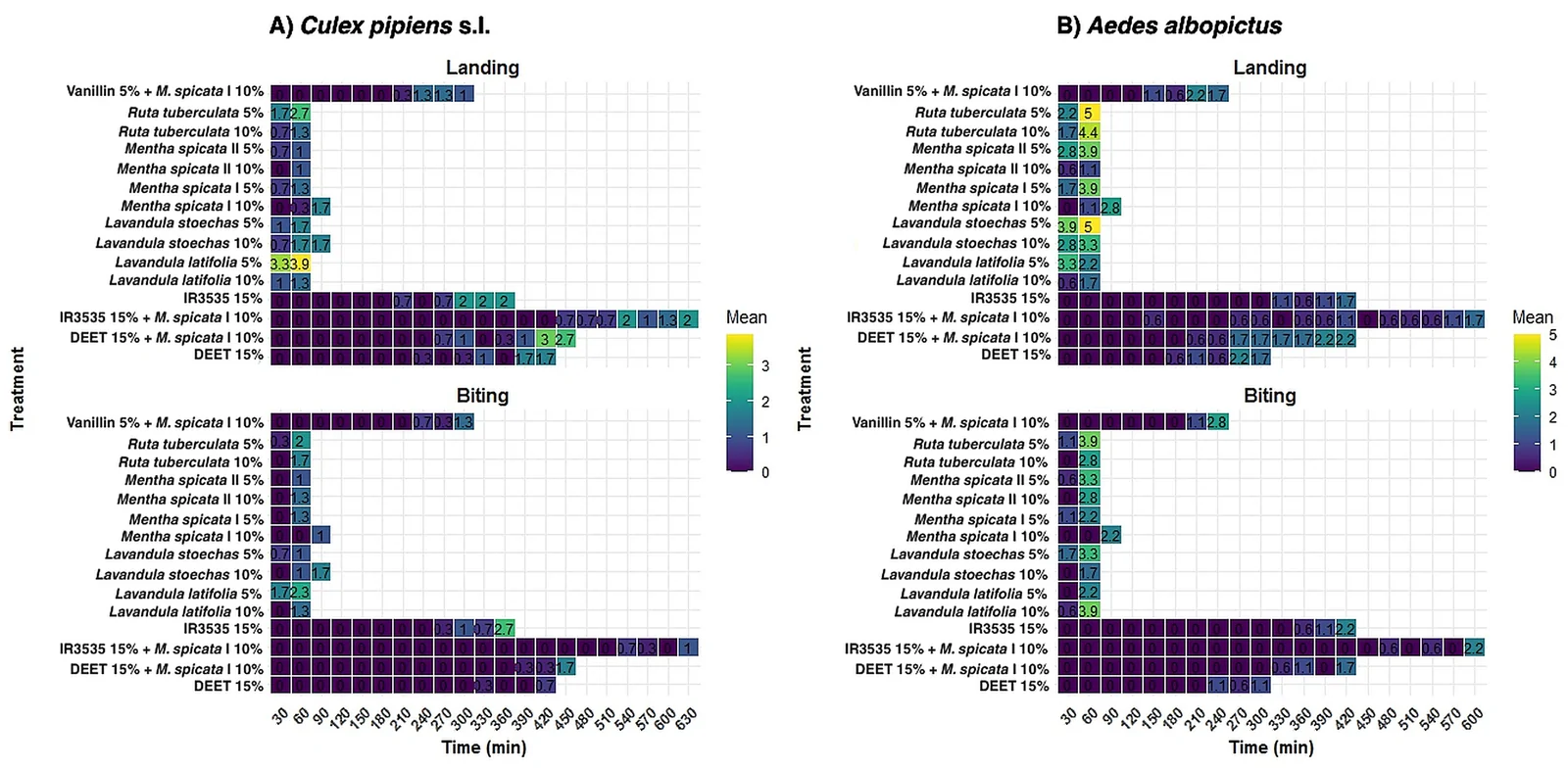
(**A**) Heatmap of mosquito behavior (mean landing % and biting % rates) over time for the different substances evaluated against *Culex pipiens* s.l. females. (**B**) Heatmap of mosquito behavior (mean landing % and biting % rates) over time for the different substances evaluated against females *Aedes albopictus*. Values are presented as mean ± SD (*n* = 3).

Examination of the landing and biting rates revealed intriguing behavioral patterns. Notably, in a 60-min repellency test, *Cx. pipiens* s.l. showed higher biting rates than landing rates on forearms treated with *R. tuberculata* and *M. spicata* II EOs at a concentration of 10% (Figure 5). Similarly, *Ae. albopictus* displayed a similar pattern toward *M. spicata* II and the spike lavender *L. latifolia* EOs, both at a concentration of 10%. These observations were comparable to the positive control IR3535 at 15% after 7 h of complete protection time CPT (Figure 5). In addition, the highest landing percentage of *Cx. pipiens* s.l. was 3.9%, observed towards *L. latifolia* 5%, and the lowest was 0.3% for *M. spicata* I EO from Ghardaïa. Likewise, the highest landing percentage of *Ae. albopictus* was 5% for *R. tuberculata* and *L. stoechas* at 5% concentration, and the lowest was 0.6% for *L. latifolia* 10%.

## 4. Discussion

In this study, EOs from five plants were screened for their repellent activity against adult females of two mosquito species *Cx. pipiens* s.l. and *Ae. albopictus*. *M. spicata* I and *R. tuberculata* were sourced from the same region (Ghardaïa, Algeria) and extracted by hydrodistillation. As the only in-house extracted oils, they were selected for further chromatographic (GC-MS) analysis. The remaining EOs, *M. spicata* II, *L. latifolia* and *L. stoechas*, were obtained commercially, each accompanied by a Certificate of Analysis (COA), and were therefore not subjected to additional chromatographic characterization in this study. The essential oil yields of *M. spicata* I and *R. tuberculata* obtained in this study aligned with previously reported values. The yield of *M. spicata* I EO corroborates research by Brahmi et al. [52], which reported a 1.1% yield in northeastern Algeria (Béjaïa). Additionally, Allali et al. [53] reported 1.3% in northwestern Algeria (Saïda). Also, Benomari et al. [54] suggested that these variations are linked to vegetative growth cycles. The percentage yield of *R. tuberculata* from Ghardaïa is comparable to those reported by Haddouchi et al. [55], who extracted EOs from four species of the *Ruta* genus in Algeria, where *R. tuberculata* was collected from Bechar, southern Algeria, and yielded 0.1%.

This research demonstrated that all five tested EOs had a repellent effect on both mosquito species, *Cx. pipiens* s.l. and *Ae. Albopictus*, where *M. spicata* I EO from Ghardaïa exhibited the most pronounced repellent efficacy. The mixture of this EO at 10% with IR3535 at 15% provided the longest CPT for both mosquito species compared to the positive control. The blend of this EO with vanillin enhanced repellency levels against both mosquito species.

Our results support earlier studies on the repellent efficacy of *M. spicata*, commonly known as spearmint. In several African countries, including Algeria, this plant has long been utilized as a medicinal plant [52]. The repellency of *M. spicata* I EO sourced from Ghardaïa was superior to that from El Bayadh against both mosquito species, *Cx. pipiens* s.l. and *Ae. albopictus*. The distinct chemical composition of the two EOs accounts for this variation. Although *M. spicata* I and II belong to the same species, the chemical profiles can vary naturally depending on geographic region, genetics, climatic, annual, or seasonal factors [24], and the levels of volatile compounds vary with phenological stages and specific plant parts [56]. Consequently, direct quantitative comparison should be interpreted with caution. However, qualitative comparison of major compound classes and correlation with observed bioactivities remains valid and provides insight into composition–activity relationships within the species. Therefore, the chemical profiling data show that both EOs share the Carvone chemotype; Carvone (-) and (S)-(+)-Carvone represent two distinct enantiomers with the same identical chemical and physical characteristics [56]. However, the existing literature suggests that a substance’s enantiomers can elicit diverse biological responses [57]. Additionally, monoterpenoids exhibit significantly lower molecular weights than sesquiterpenoids, making them more volatile. Consequently, they demonstrate heightened short-term spatial repellency, gradually diminishing to minimal levels once substantial quantities have dissipated from the applied surface [27]. Beyond the qualitative identification of major constituents, our results also suggest that mosquito repellency and CPT are influenced by the relative proportions and physicochemical properties of these compounds, particularly their volatility. Oils dominated by highly volatile monoterpenoids may provide strong initial repellency but shorter protection times, whereas compositions including less volatile oxygenated monoterpenes or aliphatic ketones such as 2-undecanone could contribute to more sustained activity on the skin. Future studies should therefore test reconstituted blends based on the present GC-MS profiles and integrate evaporation kinetics to better understand the respective contributions of individual constituents and their interactions to overall repellent performance.

These results are in line with previous findings on the repellency of *M. spicata* essential oil. Recently, Abbas et al. [16] investigated the repellency of EOs derived from two populations of *M. spicata* (I and II) against *Ae. aegypti*, *Anopheles gambiae* s.l. and *Cx. quinquefasciatus* Say. adult mosquitoes, which showed higher repellency compared to DEET after 45 and 75 min, respectively. Their repellency was observed up to 150 and 210 min against *Ae. aegypti* and *Cx. quinquefasciatus*, respectively. In a previous study, Azeem et al. [58] screened the repellent activity of seven EOs, and *M. spicata* showed the highest repellent activity against *Ae. aegypti* for up to 45 min. Similarly, Ojewumi et al. [59] demonstrated that formulations such as creams and lotions containing higher concentrations of *M. spicata* leaf oil extract can extend repellency up to 4 h, making them practical for personal protection.

The repellent properties of *L. stoechas* may be attributed to its high concentration of camphor, a monoterpene known for its repellent activity [60]. Few studies have investigated its repellent effects. Drapeau et al. [61] conducted a study of 19 EOs from Corsica. They investigated the repellent effects of *L. stoechas* on yellow fever mosquito *Ae. aegypti* using Y-tube olfactometer; *L. stoechas* was one of the most potent essential oils, yielding over 60% repellency achieving a reduction of 30% in mosquito activity compared to the control, and the authors suggested the use of this EO as a secondary agent to prolong the efficacy period of common repellents like PMD or DEET. Abdel-Baki et al. [62] indicate that *L. stoechas* EO exhibited weak repellency against *Rhipicephalus annulatus* (Say, 1821) larvae, showing only a slight effect in the first hour at the highest concentration of 10%; no significant repellent activity was observed in subsequent hours. Kayedi et al. [63] reported that *L. officinalis* showed significant repellency against *Anopheles stephensi* Liston (1901), even at low concentrations.

This is the first report on the repellency of *L. latifolia* and *R. tuberculata* EOs on *Cx. pipiens* s.l. and *Ae. albopictus* adult mosquitoes. Indeed, many studies have assessed the repellent effect of the spike lavender (*L. latifolia*) EO on stored-product pests. In this regard, Kheloul et al. [64] demonstrated the repellent effects of the spike lavender on adult *Tribolium confusum* du Val (Coleoptera: Tenebrionidae) even at highly diluted doses. According to another study, this EO was less effective as a repellent than other tested EOs against *Tribolium castaneum* (Herbst, 1797) larvae, although it showed significant insecticidal potency against *T. castaneum* adults [65]. Additionally, various *Ruta* species have been assessed for their repellent effectiveness against diverse mosquito species. Thus, the mosquito-biting-deterrent effect was reported by Ali et al. [66] when assessing the repellent activity of *Ruta chalepensis* L. EO at a concentration of 50 µg/cm^2^, which was similar to DEET at 4.8 µg/cm^2^ against *Ae. aegypti* and *Cx. quadrimaculatus* females. Moreover, the results from repellency tests using the human-bait technique highlighted that *R. chalepensis* EO from wild plants collected in Tunisia was an effective repellent against *Ae. albopictus* even at lower concentrations; this EO was able to repel 50% of mosquitoes for at least 45 min at the highest concentration of 0.08 µL/cm^2^ of skin [67].

Another factor influencing the comparability of repellent test findings is the biting pressure in the mosquito population [68]. In our study, the behavioral response of mosquitoes showed higher biting rates toward specific EOs at the highest concentration, which may be explained by various hypotheses and scenarios found in the literature. This occurrence could be used to distinguish between repellent and feeding-deterrent effects and to evaluate the efficacy of each test substance [49]. Thus, Phasomkusolsil & Soonwera [50] reported that a substance with a prolonged protection time and a low biting rate is effective as a repellent and a feeding deterrent. Conversely, if the protection time is extended but the biting rate is high, the substance is more of a repellent than a feeding deterrent. In addition, Afify & Potter [69] suggest that various mosquito species exhibit distinct behavioral responses to repellents. These biological differences may affect how each mosquito species interacts with the repellents [68]. Moreover, in studies assessing repellent effectiveness, factors resulting from people’s daily routines are nearly impossible to control [70]. The volatile compounds emitted by vertebrate skin play a pivotal role in facilitating mosquito host detection [71]. Indeed, CO_2_ is considered a synergist of skin volatiles, eliciting stronger behavioral responses in host-seeking mosquitoes than volatiles alone [71].

Nevertheless, Barnard [68] suggested that low biting pressure in the mosquito test population will lead to an overestimation of the duration of repellent protection, regardless of other factors. Therefore, Fatou & Müller [72] indicated that one major limitation of the arm-in-cage test, which has not received much attention, is that the experimental setup confines mosquitoes to a restricted area near the repellent-treated forearm, which is quite different from what a host-seeking insect would typically experience in nature. In this confined setting, indications such as the repellent qualities of volatile odorants that act over longer distances may be obscured by non-chemical signals, such as vision, heat, or humidity, which may become more important for host location than odorants. As a result, the findings from tests such as the arm-in-cage study might be influenced not only by the composition of the topical repellent but also by the circumstances under which it is offered to the host-seeking mosquito, an element that has been largely overlooked. This constitutes a limitation of our study. Thus, enhancing the understanding of how insect repellents affect mosquitoes’ olfactory systems is crucial for improving repellents [73].

## 5. Conclusions

This study highlights the significant repellent potential of Algerian native aromatic plants against *Cx. pipiens* s.l. and *Ae. albopictus*. Our results support the strategic valorization of local botanical resources as sustainable raw materials for the bio-pesticide industry, contributing to the global prevention of vector-borne diseases. While the arm-in-cage results are promising, field trials and rigorous safety assessments are essential for transitioning from laboratory screening to commercial application, as natural origin must always be accompanied by toxicological validation. Specifically, the integration of *M. spicata* I EO from Ghardaïa into hybrid formulations (with DEET or IR3535) offers a viable industrial path to enhance protection efficacy and mitigate the toxicity of isolated compounds alone. Ultimately, leveraging these aromatic plants not only promotes local agricultural biodiversity but also plays a crucial role in the global prevention of vector-borne diseases through innovative, bio-based protection.

## Figures and Tables

**Table 1:** List of plants and essential oils used in this study.

Scientific Name	Plant Origin	Common/English Name	Algerian Name	Source
*Mentha spicata* I	Ghardaïa	Spearmint	E’Naânaâ	Lab extract
*Ruta tuberculata*	Ghardaïa	Plant of the mosquito Rue	El Fijel Fidjla	Lab extract
*Mentha spicata* II	El Bayadh	Spearmint	E’Naânaâ	LEOsD
*Lavandula latifolia*	Tadmit-Djelfa	Spike Lavender Aspic Lavender	Khozama Halhal	LEOsD
*Lavandula stoechas*	Mitidja	French Lavender	Khozama Halhal	LEOsD

LEOsD: Local Essential Oil Distillery (Sarl ZIPHEE.BIO).

**Table 2:** Major compounds and CAS numbers of the commercial essential oils, according to their Certificates of Analysis (COAs). Production process: steam distillation.

Commercial EO	Major Compounds	(%)	CAS N°/Parts of the Plant
*Mentha spicata* II	Carvone (-) Limonene Eucalyptol Myrcene ß-bourbonene α-pinene Menthol ß-caryophyllene Octanol-3 Carvyl acetate	64.0 19.0 2.5 2.2 1.6 1.2 1.2 1.1 1.0 1.0	84696-51-5 Flowering tops
*Lavandula stoechas*	Fenchone Camphor Myrtenyl acetate α-pinene Camphene Limonene Linalol Eucalyptol	37.0 33.0 5.2 4.5 3.5 1.1 1.1 0.9	90063-38-0 Freshly trimmed flowering tops
*Lavandula latifolia*	Eucalyptol ß-pinene α-terpinol Camphor α-pinene Myrcene Sabinene	67.7 8.0 3.8 3.2 3.0 1.7 1.2	ND Leafy branches

ND: non-determined.

**Table 3:** An overview of the prepared dilutions.

Test Substances	Concentrations	References
*Mentha spicata* I	5%	[40,41]
	10%	
*Ruta tuberculata*	5%	[36]
	10%	
*Mentha spicata* II	5%	[40,42]
	10%	
*Lavandula latifolia*	5%	[36]
	10%	
*Lavandula stoechas*	5%	[43,44]
	10%	
DEET	15%	[4,45]
IR3535	15%	
IR3535 + *M. spicata* I	IR3535 15% + *M. spicata* I 10%	[46,47]
DEET + *M. spicata* I	DEET 15% + *M. spicata* I 10%	
Vanillin + *M. spicata* I	Vanillin 5% + *M. spicata* I 10%	

**Table 4:** Chemical composition of *Mentha spicata* I essential oil from Ghardaïa.

N°	Name	Calculated RI (Ref RI)	CAS Number	% Area
1	α-thujene	922 (924)	2867-05-2	0.2
2	α-pinene	928 (932)	7785-70-8	1.5
3	Camphene	941 (946)	79-92-5	0.1
4	Sabinene	966 (969)	3387-41-5	0.7
5	β-pinene	968 (974)	127-91-3	1.6
6	β-myrcene	985 (988)	123-35-3	0.8
7	α-phyllandrene	998 (1002)	99-83-2	0.2
8	α-terpinene	1012 (1014)	99-86-5	0.9
9	Limonene	1017 (1024)	138-86-3	36.8
10	D-limonene	1020 (/)	5989-27-5	3.0
11	cis-β-ocimene	1025 (1032)	3338-55-4	0.3
12	α-ocimene	1034 (1044)	502-99-8	0.1
13	γ-terpinene	1043 (1054)	99-85-4	1.3
14	(+)-4-carene	1070 (/)	29050-33-7	0.8
15	4-carvomenthol	1164 (1174)	562-74-3	1.5
16	cis-dihydrocarvone	1180 (1191)	3792-53-8	0.4
17	trans-dihydrocarvone	1188(1200)	5948-04-9	0.1
18	pulegone	1220 (1233)	89-82-7	0.3
19	(S)-(+)-carvone	1236 (1239)	2244-16-8	44.2
20	carvone oxide-cis	1261 (1259)	18383-49-8	0.1
21	dihydroedulan IA	1264 (/)	74006-61-4	0.1
22	dihydroedulan II	1269 (/)	41678-32-4	0.1
23	2-undecanone	1295 (1293)	112-12-9	0.6
24	trans-carveyl acetate	1341 (1339)	1134-95-8	0.2
25	β-bourbonene	1376 (1382)	5208-59-3	1.4
26	β-elemene	1386 (1389)	515-13-9	0.4
27	caryophyllene	1398 (1408)	87-44-5	1.1
28	α-amorphene	1457 (1465)	23515-88-0	0.1
29	Bicyclosesquiphellandrene	1464 (1470)	54324-03-7	0.2
30	Germacrene D	1479 (1484)	23986-74-5	0.6
31	Elixene	1503 (1511)	3242-08-8	0.1
32	1S, cis-calamenene	1521 (1528)	483-77-2	0.1
33	Caryophyllene oxide	1573 (1582)	1139-30-6	0.1
Total identified: 98.56%				

RI: Retention Index; Ref RI: Reference Retention Index.

**Table 5:** Chemical composition of *Ruta tuberculata* essential oil from Ghardaïa.

N°	Name	Calculated RI (Ref RI)	CAS Number	% Area
1	α-thujene	923 (925)	002867-05-2	1.05
2	α-pinene	928 (932)	007785-70-8	1.36
3	Camphene	942 (952)	000079-82-5	0.07
4	Sabinene	966 (974)	003387-41-5	1.46
5	β-myrcene	968 (991)	000123-35-3	2.41
6	α-phellandrene	999 (1005)	000099-83-2	1.44
7	(+)-3-carene	1005 (1011)	000498-15-7	3.25
8	α-terpinene	1012 (1017)	000099-86-5	0.53
9	p-cymene	1021 (1025)	000099-87-6	0.80
10	β-phellandrene	1025 (1031)	000555-10-2	8.85
11	β-trans-ocimene	1031 (1038)	003779-61-1	0.19
12	α-ocimene	1041 (1048)	003338-55-4	0.37
13	γ-terpinene	1042 (1050)	000099-85-4	0.22
14	terpinolene	1070 (1079)	000586-62-9	0.70
15	2-nonanone	1085 (1092)	000821-55-6	0.55
16	trans-2-menthenol	1109 (1112)	029803-81-4	5.42
17	cis-2-menthenol	1128 (1122)	029803-82-5	3.58
18	Terpinen-4-ol	1164 (1160)	000562-74-3	0.66
19	2-decanone	1176 (1172)	000693-54-9	0.50
20	Octyl acetate	1195 (1193)	000112-14-1	1.98
21	2-nonanol, acetate	1219 (1221)	014936-66-4	0.49
22	Piperitone	1236 (1233)	000089-81-6	3.17
23	1,7,7-trimethylbicyclo[2.2.1]hept-2-yl acetate	1270 (1277)	092618-89-8	0.40
24	2-undecanone	1327 (1299)	000112-12-9	45.26
25	Dihydrocarvyl acetate	1376 (1330)	20777-49-5	1.72
26	cis-geranyl acetate	1355 (1365)	000141-12-8	0.21
27	β-bourbonene	1368 (1382)	005208-59-3	0.12
28	2-dodecanone	1385 (1395)	006175-49-1	0.25
29	Caryophyllene	1402 (1419)	000087-44-5	2.43
30	γ-elemene	1417 (1432)	029873-99-2	0.27
31	2-undecanol, acetate	1421 (1433)	014936-67-5	0.54
32	Humulene	1435 (1431)	006753-98-6	0.22
33	Germacrene D	1463 (1477)	023986-74-5	0.34
34	Eremophilene	1468 (1486)	010219-75-7	0.85
35	2-tridecanone	1483 (1496)	000593-08-8	0.38
36	α-panasinsen	1498 (1527)	056633-28-4	0.22
37	Germacrene B	1537 (1550)	015423-57-1	4.93
38	Caryophyllene oxide	1562 (1574)	001139-30-6	0.62
39	Eremophilene	1596 (1486)	010219-75-7	0.17
40	Pentadecanal	1693 (1694)	002765-11-9	0.10
41	Mintsulfide	1709 (1744)	072445-42-2	0.12
42	Quinoline, 2-(1-methylethyl)-	1741 (1473)	017507-24-3	0.21
43	Piperonylcyanoacetic acid hydrazide	1741 (2260)	014731-76-1	0.22
44	α-selinene	1818 (1494)	000473-13-2	0.11
45	N/A	1836	N/A	0.10
46	9,12,15-octadecatrienal	1865 (2058)	026537-71-3	0.12
47	Psoralen, 3-(α, α-dimethylallyl)-	2165 (2211)	013164-03-9	0.88
48	Octacosane	2809 (2800)	000630-02-4	0.11
Total identified: 99.79%				

RI: Retention Index; Ref RI: Reference Retention Index.

**Table 6:** The average number of mosquito (*Culex pipiens* s.l.) landings on hands treated with the test substance and the negative control (solvent).

Test Substance	ANML-S	ANML-TS	*p* Value	df, *t*
*Mentha spicata* I 5%	15.67 ± 1.52	0.66 ± 0.57	<0.000	4, 15.90
*Mentha spicata* I 10%	16.67 ± 2.08	0.00	-	-
*Ruta tuebrculata* 5%	12.00 ± 1.00	1.66 ± 0.57	0.000	4, 15.50
*Ruta tuebrculata* 10%	13.33 ± 1.52	0.66 ± 0.57	0.000	4, 13.43
*Lavandula latifolia* 5%	17.00 ± 1.00	3.33 ± 1.67	0.000	4, 12.16
*Lavandula latifolia* 10%	12.66 ± 3.05	1.00 ± 0.00	-	-
*Mentha spicata* II 5%	12.00 ± 1.00	1.66 ± 0.57	0.000	4, 15.50
*Mentha spicata* II 10%	13.33 ± 1.52	0.66 ± 0.57	0.000	4, 13.43
*Lavandula stoechas* 5%	14.64 ± 2.64	1.00 ± 0.00	-	-
*Lavandula stoechas* 10%	12.00 ± 2.64	0.66 ± 0.57	0.002	4, 7.24
DEET 15%	14.66 ± 3.05	0.00 ± 0.00	-	-
IR3535 15%	23.33 ± 1.52	0.00 ± 0.00	-	-
IR3535 15% + *Mentha spicata* I 10%	18.00 ± 2.00	0.00 ± 0.00	-	-
DEET 15% + *Mentha spicata* I 10%	18.66 ± 1.52	0.00 ± 0.00	-	-
Vanillin 5% + *Mentha spicata* I 10%	11.33 ± 1.52	0.00 ± 0.00	-	-

ANML-S: average number of mosquito landings on the hand treated with solvent (negative control); ANML-TS: average number of mosquito landings on the hand treated with the test substance; each treatment was compared separately with the negative control using an unpaired Student’s *t*-test. The test statistic (*t*), degrees of freedom (df), and corresponding *p*-value are reported for each comparison. Statistical significance was considered at *p* < 0.05; (-): statistical comparisons were not performed for treatments with zero mosquito landings after application because the post-treatment data exhibited no variability (all observations = 0), preventing meaningful inferential statistical analysis.

**Table 7:** The average number of mosquito (*Aedes albopictus*) landings on hands treated with the test substance and the negative control (solvent).

Test Substance	ANML-S	ANML-TS	*p* Value	df, *t*
*Mentha spicata* I 5%	17.00 ± 2.00	1.66 ± 0.00	-	-
*Mentha spicata* I 10%	18.00 ± 2.64	0.00	-	-
*Ruta tuebrculata* 5%	16.66 ± 1.52	1.10 ± 0.95	0.000	4, 14.95
*Ruta tuebrculata* 10%	15.00 ± 2.64	1.66 ± 0.00	-	-
*Lavandula latifolia* 5%	12.33 ± 1.52	3.33 ± 1.67	0.002	4, 6.89
*Lavandula latifolia* 10%	13.00 ± 2.00	0.55 ± 0.95	0.001	4, 9.72
*Mentha spicata* II 5%	12.00 ± 1.73	2.77 ± 0.96	0.001	4, 8.06
*Mentha spicata* II 10%	10.33 ± 0.57	0.00	-	-
*Lavandula stoechas* 5%	12.20 ± 2.64	3.88 ± 0.96	0.003	4, 6.32
*Lavandula stoechas* 10%	10.66 ± 1.15	2.77 ± 0.96	0.001	4, 9.53
DEET 15%	16.66 ± 1.52	0.00 ± 0.00	-	-
IR3535 15%	15.00 ± 2.00	0.00 ± 0.00	-	-
IR3535 15% + *Mentha spicata* I 10%	17.33 ± 3.05	0.00 ± 0.00	-	-
DEET 15% + *Mentha spicata* I 10%	16.66 ± 3.78	0.00 ± 0.00	-	-
Vanillin 5% + *Mentha spicata* I 10%	14.66 ± 1.52	0.00 ± 0.00	-	-

ANML-S: average number of mosquito landings on the hand treated with solvent (negative control); ANML-TS: average number of mosquito landings on the hand treated with the test substance; each treatment was compared separately with the negative control using an unpaired Student’s *t*-test. The test statistic (*t*), degrees of freedom (df), and corresponding *p*-value are reported for each comparison. Statistical significance was considered at *p* < 0.05; (-): statistical comparisons were not performed for treatments with zero mosquito landings after application because the post-treatment data exhibited no variability (all observations = 0), preventing meaningful inferential statistical analysis.

## Data Availability

The raw data supporting the conclusions of this article will be made available by the authors on request.

## Abbreviations

The following abbreviations are used in this manuscript:

EOs	Essential Oils
AIC	Arm In Cage
CPT	Complete Protection Time

## References

[1] Lucas K.J., Myles K.M., Raikhel A.S. (2013). Small RNAs: A new frontier in mosquito biology. Trends Parasitol..

[2] World Health Organization Vector-Borne Diseases Fact Sheet. https://www.who.int/news-room/fact-sheets/detail/vector-borne-diseases.

[3] Bonizzoni M., Gasperi G., Chen X., James A.A. (2013). The invasive mosquito species *Aedes albopictus*: Current knowledge and future perspectives. Trends Parasitol..

[4] Barnard D.R., Xue R.D. (2004). Laboratory Evaluation of Mosquito Repellents Against *Aedes albopictus*, *Culex nigripalpus*, and *Ochlerotatus triseriatus* (Diptera: Culicidae). J. Med. Entomol..

[5] Yadouleton A., Hounkanrin G., Tchibozo C., Bialonski A., Schmidt-Chanasit J., Jöst H. (2022). First Detection of the Invasive Mosquito Vector *Aedes albopictus* (Diptera: Culicidae) in Benin, West Africa. J. Med. Entomol..

[6] Barman S., Semenza J.C., Singh P., Sjödin H., Rocklöv J., Wallin J. (2025). A Climate and population dependent diffusion model forecasts the spread of *Aedes albopictus* mosquitoes in Europe. Commun. Earth Environ..

[7] Izri A., Bitam I., Charrel R.N. (2011). First entomological documentation of *Aedes* (Stegomyia) *albopictus* (Skuse, 1894) in Algeria. Clin. Microbiol. Infect..

[8] Benallal K.E., Garni R., Bouiba L., Harrat Z. (2019). First Detection of *Aedes* (*Stegomyia*) *albopictus* (Diptera: Culicidae) in Algiers, the Capital City of Algeria. J. Arthropod-Borne Dis..

[9] Hamaidia K., Soltani N. (2021). Short communication: New report of *Aedes albopictus* in Souk Ahras, Northeast Algeria. Biodiversitas J. Biol. Divers..

[10] Arroussi R., Bouaziz A., Boudjelida H. (2021). Mosquito survey reveals the first record of *Aedes* (Diptera: Culicidae) species in urban area, Annaba district, Northeastern Algeria. Pol. J. Entomol..

[11] Korba R.A., Alayat M.S., Bouiba L., Boudrissa A., Bouslama Z., Boukraa S., Francis F., Failloux A.-B., Boubidi S.C. (2016). Ecological differentiation of members of the *Culex pipiens* complex, potential vectors of West Nile virus and Rift Valley fever virus in Algeria. Parasit. Vectors.

[12] Hamaidia H., Berchi S. (2018). Etude systématique et écologique des Moustiques (Diptera: Culicidae) dans la région de Souk-Ahras (Algérie). Faun. Entomol..

[13] Hafsi N.H., Hamaidia K., Barour C., Soltani N. (2021). A survey of Culicidae (Insecta Diptera) in some habitats in Souk-Ahras province (Northeast Algeria). Biodiver. J..

[14] Rouibi A., Rouibi A., Rouibi A. (2024). The First Culicidae Inventory in the Region of Guelma (Northeast Algeria). Transylv. Rev. Syst. Ecol. Res..

[15] Amraoui F., Krida G., Bouattour A., Rhim A., Jabeur D., Harrat Z., Boubidi S.-C., Tijane M., Sarih M., Failloux A.-B. (2012). *Culex pipiens*, an Experimental Efficient Vector of West Nile and Rift Valley Fever Viruses in the Maghreb Region. PLoS ONE.

[16] Abbas M.G., Binyameen M., Azeem M., Majeed S., Sarwar Z.M., Nazir A., Sharif M.M.I., Parveen A., Mozūratis R. (2025). Chemical analysis, repellent, larvicidal, and oviposition deterrent activities of plant essential oils against *Aedes aegypti*, *Anopheles gambiae*, and *Culex quinquefasciatus*. Front. Insect Sci..

[17] Wang Z., Song J., Chen J., Song Z., Shang S., Jiang Z., Han Z. (2008). QSAR study of mosquito repellents from terpenoid with a six-member-ring. Bioorg. Med. Chem. Lett..

[18] Sharma M., Ajazuddin, Nagori K., Jain V., Balan N.S. (2023). Herbal, Safe and effective Mosquito repellents: Recent Development and Opportunity. Res. J. Pharm. Technol..

[19] Noguera-Gahona M., Peña-Moreno C., Quiñones-Sobarzo N., Weinstein-Oppenheimer C., Guerra-Zúñiga M., Collao-Ferrada X. (2025). Repellents against *Aedes aegypti* bites: Synthetic and natural origins. Front. Insect Sci..

[20] Mapossa A.B., Focke W.W., Tewo R.K., Androsch R., Kruger T. (2021). Mosquito-repellent controlled-release formulations for fighting infectious diseases. Malar. J..

[21] Moreno-Gómez M., Bueno-Marí R., Carr B.T., Bowman G.R., Faherty G.W., Gobbi C., Palm J.M., Van Sloun P., Miranda M.Á. (2021). Two New Alternatives to the Conventional Arm-in-Cage Test for Assessing Topical Repellents. J. Med. Entomol..

[22] Şengül Demirak M.Ş., Canpolat E. (2022). Plant-Based Bioinsecticides for Mosquito Control: Impact on Insecticide Resistance and Disease Transmission. Insects.

[23] De Souza M.A., Da Silva L., Macêdo M.J.F., Lacerda-Neto L.J., Dos Santos M.A.C., Coutinho H.D.M., Cunha F.A.B. (2019). Adulticide and repellent activity of essential oils against *Aedes aegypti* (Diptera: Culicidae)—A review. S. Afr. J. Bot..

[24] Koul O., Walia S., Dhaliwal G.S. (2008). Essential Oils as Green Pesticides: Potential and Constraints. Biopestic. Int..

[25] Kamaraj C., Satish Kumar R.C., Al-Ghanim K.A., Nicoletti M., Sathiyamoorthy V., Sarvesh S., Ragavendran C., Govindarajan M. (2023). Novel Essential Oils Blend as a Repellent and Toxic Agent against Disease-Transmitting Mosquitoes. Toxics.

[26] Patel K., Paroha S., Patani P. (2024). Scent and defense: Dual function encapsulated oil, a combined novel approach for perfume and mosquito repellent. J. Pop. Ther. Clin. Pharmacol..

[27] Da Silva M.R.M., Ricci-Junior E. (2020). An approach to natural insect repellent formulations: From basic research to technological development. Acta Trop..

[28] Carroll S.P., Loye J. (2006). PMD, a Registered Botanical Mosquito Repellent with Deet-Like Efficacy. J. Am. Mosq. Control Assoc..

[29] Meddour R., Sahar O., Jury S. (2023). New analysis of the endemic vascular plants of Algeria, their diversity, distribution pattern and conservation status. Willdenowia.

[30] Cheraif K., Bakchiche B., Gherib A., Bardaweel S.K., Çol Ayvaz M., Flamini G., Ascrizzi R., Ghareeb M.A. (2020). Chemical Composition, Antioxidant, Anti-Tyrosinase, Anti-Cholinesterase and Cytotoxic Activities of Essential Oils of Six Algerian Plants. Molecules.

[31] Aribi L., Bounechada M., Khenchouche A., Nabti I., Bensebaa F., Boudechicha A. (2024). Phytochemical composition and larvicidal activity of *Saccocalyx satureioides* Coss. et Durieu essential oil against *Culex pipiens* S.L. and *Culiseta longiareolata* (Diptera: Culicidae). Nat. Resour. Sustain. Dev..

[32] Ammar S., Noui H., Djamel S., Madani S., Maggi F., Bruno M., Romano D., Canale A., Pavela R., Benelli G. (2020). Essential oils from three Algerian medicinal plants (*Artemisia campestris*, *Pulicaria arabica*, and *Saccocalyx satureioides*) as new botanical insecticides?. Environ. Sci. Pollut. Res..

[33] Nabti I., Bounechada M. (2019). Larvicidal Activities of Essential Oils Extracted from Five Algerian Medicinal Plants against *Culiseta longiareolata* Macquart. Larvae (Diptera Culicidae). Eur. J. Biol..

[34] Bouguerra N., Djebbar F.T., Soltani N. (2017). *Algerian Thymus vulgaris* essential oil: Chemical composition and larvicidal activity against the mosquito *Culex pipiens*. Int. J. Mosq. Res..

[35] Rehman J.U., Ali A., Khan I.A. (2014). Plant based products: Use and development as repellents against mosquitoes: A review. Fitoterapia.

[36] Bedini S., Flamini G., Ascrizzi R., Venturi F., Ferroni G., Bader A., Girardi J., Conti B. (2018). Essential oils sensory quality and their bioactivity against the mosquito *Aedes albopictus*. Sci. Rep..

[37] Barbouchi M., Benzidia B., Choukrad M. (2021). Chemical variability in essential oils isolated from roots, stems, leaves and flowers of three *Ruta* species growing in Morocco. J. King Saud Univ. Sci..

[38] Adams R.P. (2007). Identification of Essential Oil Components by Gas Chromatography/Mass Spectrometry.

[39] World Health Organization Guidelines for Efficacy Testing of Mosquito Repellents for Human Skin. https://www.who.int/publications/i/item/WHO-HTM-NTD-WHOPES-2009.4.

[40] Lopez A.D., Whyms S., Luker H.A., Galvan C., Holguin F.O., Hansen I.A. (2025). Repellency of Essential Oils and Plant-Derived Compounds Against *Aedes aegypti* Mosquitoes. Insects.

[41] Luker H.A., Salas K.R., Esmaeili D., Holguin F., Bendzus-Mendoza H., Hansen I. (2023). Repellent efficacy of 20 essential oils on *Aedes aegypti* mosquitoes and *Ixodes scapularis* ticks in contact-repellency assays. Sci. Rep..

[42] Lalthazuali, Mathew N. (2017). Mosquito repellent activity of volatile oils from selected aromatic plants. Parasitol. Res..

[43] Asadollahi A., Khoobdel M., Zahraei-Ramazani A., Azarmi S., Mosawi S.H. (2019). Effectiveness of plant-based repellents against different *Anopheles* species: A systematic review. Malar. J..

[44] Trongtokit Y., Rongsriyam Y., Komalamisra N., Apiwathnasorn C. (2005). Comparative repellency of 38 essential oils against mosquito bites. Phytother. Res..

[45] Lupi E., Hatz C., Schlagenhauf P. (2013). The efficacy of repellents against *Aedes*, *Anopheles*, *Culex* and *Ixodes* spp.—A literature review. Travel Med. Infect. Dis..

[46] Lin C.-X., Huang X., Sun Y.-H., Lan B.-H., Deng A.-Q., Chen L.-Y., Lin Q.-Y., Huang X.-T., Li J.-L., Wu C. (2026). Triple-Olfactory Mechanism Synergy: Development of a Long-Lasting DEET–Botanical Composite Repellent Against *Aedes albopictus*. Insects.

[47] Manh H., Tuyet O.T. (2020). Larvicidal and Repellent Activity of *Mentha arvensis* L. Essential Oil against *Aedes Aegypti*. Insects.

[48] Ahmad I., Amalia R., Yusmalinar S. (2025). Effectiveness and Public Perception of Synthetic and Natural-Based Mosquito Repellents Against *Aedes aegypti* in Indonesia. 3BIO J. Biol. Sci. Technol. Manag..

[49] Amer A., Mehlhorn H. (2006). Repellency effect of forty-one essential oils against *Aedes*, *Anopheles*, and *Culex* mosquitoes. Parasitol. Res..

[50] Phasomkusolsil S., Soonwera M. (2010). Insect repellent activity of medicinal plant oils against *Aedes aegypti* (Linn.), *Anopheles minimus* (Theobald) and *Culex quinquefasciatus* Say based on protection time and biting rate. Southeast Asian J. Trop. Med. Public Health.

[51] R Core Team (2025). R: A Language and Environment for Statistical Computing, Version 4.5.1.

[52] Brahmi F., Adjaoud A., Marongiu B., Falconieri D., Yalaoui-Guellal D., Madani K., Chibane M. (2016). Chemical and biological profiles of essential oils from *Mentha spicata* L. leaf from Bejaia in Algeria. J. Essent. Oil Res..

[53] Allali H., Chikhi I., Dib M.E., Muselli A., Fekih N., Meliani N., Kamal M.A., Tabti B., Costa J. (2013). Antioxidant activity and chemical analysis of *Mentha spicata* cultivated from west northern region of Algeria by headspace solid phase micro-extraction and hydro-distillation. Nat. Prod. Indian J..

[54] Benomari F.Z., Sarazin M., Chaib D., Pichette A., Boumghar H. (2023). Chemical Variability and Chemotype Concept of Essential Oils from Algerian Wild Plants. Molecules.

[55] Haddouchi F., Chaouche T.M., Zaouali Y., Ksouri R., Attou A., Benmansour A. (2013). Chemical composition and antimicrobial activity of the essential oils from four *Ruta* species growing in Algeria. Food Chem..

[56] Bouyahya A., Mechchate H., Benali T., Ghchime R., Charfi S., Balahbib A., Burkov P., Shariati M.A., Lorenzo J.M., El Omari N. (2021). Health Benefits and Pharmacological Properties of Carvone. Biomolecules.

[57] Aggarwal K.K., Khanuja S.P.S., Ahmad A., Santha Kumar T.R., Gupta V.K., Kumar S. (2002). Antimicrobial activity profiles of the two enantiomers of limonene and carvone isolated from the oils of *Mentha spicata* and *Anethum sowa*. Flavour Fragr. J..

[58] Azeem M., Zaman T., Tahir M., Haris A., Iqbal Z., Binyameen M., Nazir A., Shad S.A., Majeed S., Mozūraitis R. (2019). Chemical composition and repellent activity of native plants essential oils against dengue mosquito, *Aedes aegypti*. Ind. Crops Prod..

[59] Ojewumi M.E., Adedokun S.O., Omodara O.J., Oyeniyi E.A., Taiwo O.S., Ojewumi E.O. (2017). Phytochemical and Antimicrobial Activities of the Leaf Oil Extract of *Mentha spicata* and its Efficacy in Repelling Mosquito. Int. J. Pharm. Res. Allied Sci..

[60] Gillij Y.G., Gleiser R.M., Zygadlo J.A. (2008). Mosquito repellent activity of essential oils of aromatic plants growing in Argentina. Bioresour. Technol..

[61] Drapeau J., Fröhler C., Touraud D., Kröckel U., Geier M., Rose A., Kunz W. (2009). Repellent studies with *Aedes aegypti* mosquitoes and human olfactory tests on 19 essential oils from Corsica, France. Flavour Fragr. J..

[62] Abdel-Baki A.A.S., Aboelhadid S.M., Al-Quraishy S., Hassan A.O., Daferera D., Sokmen A., Kamel A.A. (2023). Cytotoxic, Scolicidal, and Insecticidal Activities of *Lavandula stoechas* Essential Oil. Separations.

[63] Kayedi M.H., Haghdoost A.A., Salehnia A., Khamisabadi K. (2014). Evaluation of Repellency Effect of Essential Oils of *Satureja khuzestanica* (Carvacrol), *Myrtus communis* (Myrtle), *Lavendula officinalis* and *Salvia sclarea* using Standard WHO Repellency Tests. J. Arthropod-Borne Dis..

[64] Kheloul L., Kellouche A., Bréard D., Gadenne C., Anton S. (2019). Trade-off between attraction to aggregation pheromones and repellent effects of spike lavender essential oil and its main constituent linalool in the flour beetle *Tribolium confusum*. Entomol. Exp. Appl..

[65] Sharmin R., Haque S., Rumpa M.J.F., Faruki S.I. (2024). Mortality and Repellency Effect of Some Essential Oils against *Tribolium castaneum* (Coleoptera: Tenebrionidae). Asian J. Res. Zool..

[66] Ali A., Demirci B., Kiyan H.T., Bernier U.R., Tsikolia M., Wedge D.E., Khan I.A., Başer K.H.C., Tabanca N. (2013). Biting Deterrence, Repellency, and Larvicidal Activity of *Ruta chalepensis* (Sapindales: Rutaceae) Essential Oil and Its Major Individual Constituents Against Mosquitoes. J. Med. Entomol..

[67] Conti B., Leonardi M., Pistelli L., Profeti R., Ouerghemmi I., Benelli G. (2013). Larvicidal and repellent activity of essential oils from wild and cultivated *Ruta chalepensis* L. (Rutaceae) against *Aedes albopictus* Skuse (Diptera: Culicidae), an arbovirus vector. Parasitol. Res..

[68] Barnard D. (1999). Repellency of essential oils to mosquitoes (Diptera: Culicidae). J. Med. Entomol..

[69] Afify A., Potter C.J. (2020). Insect repellents mediate species-specific olfactory behaviours in mosquitoes. Malar. J..

[70] Moreno-Gómez M., Monsonís-Güell E., Abril S., Manzanares-Sierra A., Mayol-Pérez J. (2025). Robustness of insect repellent efficacy research: Exploring the impacts of sampling design and participant heterogeneity. J. Eur. Mosq. Control Assoc..

[71] Liu F., Coutinho-Abreu I.V., Raban R., Thuy T., Dimas A.R., Merriman J.A., Akbari O.S. (2024). Engineered skin microbiome reduces mosquito attraction to mice. Proc. Natl. Acad. Sci. Nexus.

[72] Fatou M., Müller P. (2024). In the arm-in-cage test, topical repellents activate mosquitoes to disengage upon contact instead of repelling them at distance. Sci. Rep..

[73] Afify A., Betz J.F., Riabinina O., Lahondère C., Potter C.J. (2019). Commonly Used Insect Repellents Hide Human Odors from *Anopheles* Mosquitoes. Curr. Biol..

